# Taming the chaos?! Using eXplainable Artificial Intelligence (XAI) to tackle the complexity in mental health research

**DOI:** 10.1007/s00787-021-01836-0

**Published:** 2021-07-08

**Authors:** Veit Roessner, Josefine Rothe, Gregor Kohls, Georg Schomerus, Stefan Ehrlich, Christian Beste

**Affiliations:** 1 Department of Child and Adolescent Psychiatry, Faculty of Medicine, TU Dresden, Fetscherstrasse 74, 01307 Dresden, Germany; 2 Department of Psychiatry, Leipzig University Medical Center, Semmelweisstr. 10, 04103 Leipzig, Germany; 3 Division of Psychological and Social Medicine and Developmental Neurosciences, Faculty of Medicine, TU Dresden, Fetscherstrasse 74, 01307 Dresden, Germany

Mental disorders cause a significant degree of burden to affected individuals and to society at large. Reasons for this are their high prevalence (one in every two people suffers from a mental disorder at some point in their lifetime), their usually early onset (three in four patients fall ill before the age of 23), and-particularly if left untreated-their mostly chronic course, precipitating numerous disease-related disabilities and poor health outcomes [1–3]. In addition, a substantial percentage of non-responders and non-compliant patients exists. Notably, particularly for youth under the age of 18, access to diagnosis, prevention/intervention, and care for mental health problems is still relatively limited compared to common somatic issues [4]. In the following, we will explain the reason for this discrepancy and provide a possible solution.

In general, the combination of clinical and translational science within medicine has steadily increased over the years, and this has led to tremendous progress also in mental healthcare [5, 6]. Nevertheless, mental disorders are very heterogeneous, dynamic, and multi-causal phenomena. Despite the widespread recognition of their inherent complex nature (including gene-environment and psyche-soma interactions as well as developmental and other experience-based changes over the life span), progress in understanding mental disorders as multifaceted bio-psycho-social conditions remains rather slow. In addition, even if acknowledged as such, our knowledge about etiopathophysiology, diagnosis, and management of mental disorders is still incomplete. The reasons that we still lack a more comprehensive picture of mental disorders are manifold, including the dichotomy of hypothesis-driven versus exploratory data-driven research methods and resulting findings, which all have their own pros and cons [7].

Furthermore, there is still an ongoing discussion to what extent the classification systems, such as DSM or ICD, are valid for diagnosing mental disorders [8]. For example, despite great research efforts clinically usable biomarkers that could potentially improve the early identification of mental disorders or that could be utilized for early intervention strategies (e.g., as predictors for treatment response) are still lacking. Among other reasons, the nosological classification systems primarily define mental disorders categorically according to a set of core symptoms, thereby neglecting the substantial dimensional, multifactorial, and heterogeneous clinical presentation and emergence of mental disorders as well as their symptomatic overlap. Consequently, dimensional transdiagnostic approaches have been introduced into the research arena, including the Research Domain Criteria (RDoC) project [9], but these approaches are still in their infancy, and they are no less hotly debated than the “traditional” ones [10].

This increase in complexity is accompanied by substantial technological and methodological advances in the areas of (epi)genetics, neuroimaging, psychophysiology, and others. However, these highly promising new leads that each attempts to identify the (neuro)biological correlates of mental disorders-or specific valid disease subtypes-have yet failed to convincingly resolve the issue of within-and across-disorder heterogeneity as they often lack specificity [8]. For instance, despite thorough research in the area of neuroimaging, with the exception of a few and relatively rare conditions, we do not have a single indicator available grounded in brain biology that can reliably distinguish patients with a specific mental disorder from typical controls, let alone differentiate between different mental disorders or their subtypes.

**Fig. 1:**
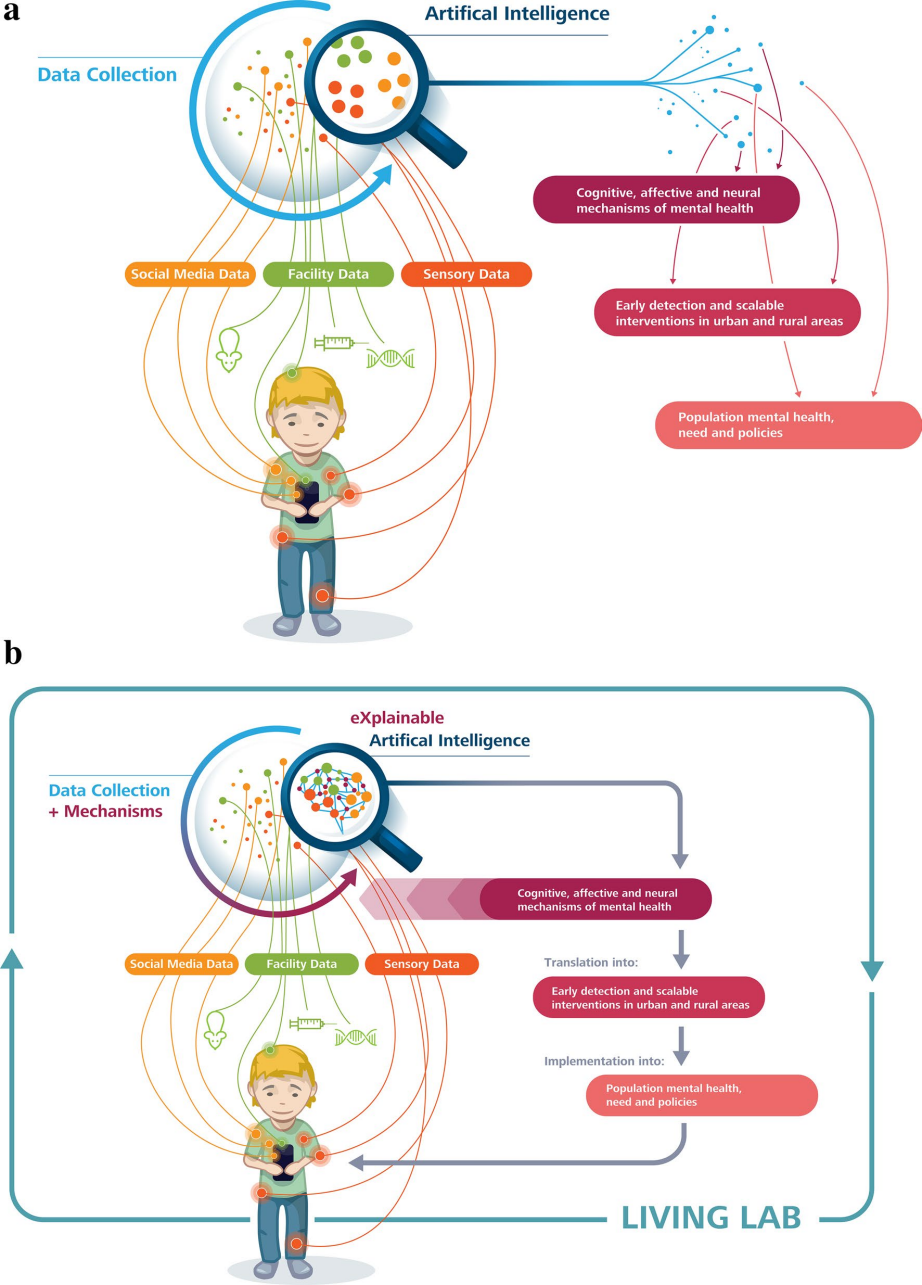
**a** Existing mental healthcare and prevention approaches using ML/AI often suffer from their lack of explainability, leading to single pieces of a puzzle, but not a meaningful picture. **b** Future holistic mental healthcare and prevention approaches (see text for details): should be based on data collection using social media data, (health) facility data, and real-time monitoring of human sensory data-analyzed by using eXplainable Artificial Intelligence (XAI)-that is guided by evidence about underlying mechanisms; and this will help to develop, implement, evaluate and optimize scalable healthcare and prevention/intervention approaches, including “classic” Health as well as eHealth and mHealth, as part of a living lab approach

Technical innovations have also led to an increased quantity and diversity of data that can be measured to help disentangling the complex nature of mental disorders. In addition to “classic” data sets from questionnaires, performance tests, and clinical interviews, three sources of data are particularly crucial in modern mental health research [11]: (1) social media data (e.g., content and color analytics of social network usage); (2) facility data (e.g., electronic health records from different digital health information systems, but also data from animal models, or genetics); and (3) sensory data (e.g., real-time monitoring of human physiological measures, such as glucose, or heart rate; see Fig. 1).

Nevertheless, collecting these diverse sets of data alone—even if they are high in quality, quantity, and ideally in validity, will not substantially improve our understanding of mental disorders as multifaceted bio-psycho-social conditions. To achieve such aim sophisticated and meaningful analytic approaches are required, including, amongst others, Machine Learning (ML)/Artificial Intelligence (AI).

The unique potential of ML/AI for characterizing complex data structures has already been successfully demonstrated in the context of neuroscientific research [12]. For example, the identification of relevant information from EEG patterns for neural classification of brain functionality has been significantly improved by ML techniques [13]. Within child and adolescent psychiatry, ML has been used, for instance, to predict the risk of psychosis [14, 15]. However, even though complex classification patterns can be identified using such exploratory data-driven ML/AI approaches, these patterns are only partially useful, because often researchers do not obtain knowledge of their internal workings (concept of a "black box" in ML/AI), and therefore, the meaning and relevance of the results can rarely be explained (but there are actually several attempts to solve this problem, e.g., [16]). This might be the main reason why the use of these approaches in the field of mental disorders (i.e., computational psychiatry) has not yet been more fruitful although their potential is obvious [7].

By contrast, eXplainable AI (XAI) methods follow the three principles transparency, interpretability and explainability. Thereby, it is possible to examine which feature(s) in the data set contribute(s) most to a specific classification pattern. One such approach is to use saliency maps [17], which are designed to visualize the relative weight or importance of feature(s) in the data that are intuitively fed into deep learning algorithms (e.g., allotting values between zero and one denoting the importance of a feature). Although XAI approaches per se cannot provide causal mechanistic insights into how the brain accomplishes a particular function or complex behaviors [7], both experimental studies on mechanisms (i.e., cognitive, affective, and neural mechanistic models of mental health) combined with results of XAI can be related to multi-modal data (e.g., EEG [17], neuroimaging, clinical, and environmental data [18]). By harvesting predictive inter-relationships among different data types, these approaches are able to outperform unimodal data models in terms of classification accuracy. This offers new opportunities, for instance, for diagnostic purposes in the realm of mental disorders in children and beyond. In particular, using XAI with everyday social media, (health) facility, and sensory data (see above) would clearly advance our understanding of the mechanisms from mental health to disorders that will help predicting risk and disease trajectories which then would allow developing personalized and scalable detection and prevention/intervention tools (e.g., eHealth and mHealth). Consequently, this would move the field forward to a transdisciplinary, integrative, context-sensitive and person-centered healthcare model [5].

However, to develop such mental healthcare model(s) even further the entire translational continuum spanning from basic science discovery, early human studies, clinical trials, implementation, evaluation, and optimization in practice and communities is required [6, 19]. For example, in attention deficit hyperactivity disorder (ADHD), basic cognitive neuroscience research, including results of EEG studies, is on its way to provide more scalable interventions, such as optimized neurofeedback training, addressing the gap between basic biomedical research and mental healthcare. Moreover, the application of neurofeedback@home brings healthcare innovations deeper into the community, including both urban and rural areas.

In contrast to the “traditional” professionally driven, more one-directional translational continuum, where scientists develop diagnostic and intervention tools for clinicians, who in turn use them in their patients, several continuous feedback loops as part of a living lab approach are needed [20]. Such feedback loops help to constantly re-adjust ongoing research priorities. The concept of living labs describes a user-centered environment for open innovation that combines multiple methods with participatory research in a real-world setting. Research particularly on child and adolescent mental health has to be closely connected to the wider community it serves to implement easy-to-access help for target groups both in urban and rural contexts, support self-management and help young people build and utilize local support networks. In this context utilizing locally established partnerships and networks as well as performing an adaptive research process with constant stakeholder input and feedback is pivotal to both the protection and the improvement of the mental health of children and adolescents in a changing social environment, particularly during critical developmental phases, including the transition from childhood to adolescence into adulthood. This altogether will help to establish models of close collaboration between academia, regional and (inter)national partners to deliver cutting-edge research, innovative clinical services, evidence-based training, and policy development that will ensure continuous improvement in the access to diagnosis, prevention/intervention and care provided to young people suffering from impairing mental disorders.
